# Migration towards SDF-1 selects angiogenin-expressing bone marrow monocytes endowed with cardiac reparative activity in patients with previous myocardial infarction

**DOI:** 10.1186/s13287-015-0028-y

**Published:** 2015-04-11

**Authors:** Raimondo Ascione, Jonathan Rowlinson, Elisa Avolio, Rajesh Katare, Marco Meloni, Helen L Spencer, Giuseppe Mangialardi, Caroline Norris, Nicolle Kränkel, Gaia Spinetti, Costanza Emanueli, Paolo Madeddu

**Affiliations:** Bristol Heart Institute, School of Clinical Sciences, University of Bristol, Upper Maudlin Road, Bristol, BS2 8HW UK; Charité – Universitätsmedizin Berlin, Berlin, Germany; IRCCS MultiMedica, Milan, Italy

## Abstract

**Introduction:**

Chemokine-directed migration is crucial for homing of regenerative cells to the infarcted heart and correlates with outcomes of cell therapy trials. Hence, transplantation of chemokine-responsive bone marrow cells may be ideal for treatment of myocardial ischemia. To verify the therapeutic activity of bone marrow mononuclear cells (BM-MNCs) selected by *in vitro* migration towards the chemokine stromal cell-derived factor-1 (SDF-1) in a mouse model of myocardial infarction (MI), we used BM-MNCs from patients with previous large MI recruited in the TransACT-1&2 cell therapy trials.

**Methods:**

Unfractioned BM-MNCs, SDF-1-responsive, and SDF-1-nonresponsive BM-MNCs isolated by patients recruited in the TransACT-1&2 cell therapy trials were tested in Matrigel assay to evaluate angiogenic potential. Secretome and antigenic profile were characterized by flow cytometry. Angiogenin expression was measured by RT-PCR. Cells groups were also intramyocardially injected in an *in vivo* model of MI (8-week-old immune deficient CD1-FOXN1^nu/nu^ mice). Echocardiography and hemodynamic measurements were performed before and at 14 days post-MI. Arterioles and capillaries density, infiltration of inflammatory cells, interstitial fibrosis, and cardiomyocyte proliferation and apoptosis were assessed by immunohistochemistry.

**Results:**

*In vitro* migration enriched for monocytes, while CD34^+^ and CD133^+^ cells and T lymphocytes remained mainly confined in the non-migrated fraction. Unfractioned total BM-MNCs promoted angiogenesis on Matrigel more efficiently than migrated or non-migrated cells. In mice with induced MI, intramyocardial injection of unfractionated or migrated BM-MNCs was more effective in preserving cardiac contractility and pressure indexes than vehicle or non-migrated BM-MNCs. Moreover, unfractioned BM-MNCs enhanced neovascularization, whereas the migrated fraction was unique in reducing the infarct size and interstitial fibrosis. *In vitro* studies on isolated cardiomyocytes suggest participation of angiogenin, a secreted ribonuclease that inhibits protein translation under stress conditions, in promotion of cardiomyocyte survival by migrated BM-MNCs.

**Conclusions:**

Transplantation of bone marrow cells helps post-MI healing through distinct actions on vascular cells and cardiomyocytes. In addition, the SDF-1-responsive fraction is enriched with angiogenin-expressing monocytes, which may improve cardiac recovery through activation of cardiomyocyte response to stress. Identification of factors linking migratory and therapeutic outcomes could help refine regenerative approaches.

**Electronic supplementary material:**

The online version of this article (doi:10.1186/s13287-015-0028-y) contains supplementary material, which is available to authorized users.

## Introduction

Bone marrow mononuclear cells (BM-MNCs) are predominant in cell therapy trials of myocardial infarction (MI) and heart failure [1]. Recent reviews and meta-analyses indicate that BM cell therapy is safe and leads to tangible improvements in cardiac function, ventricular remodeling and clinical outcomes, including incidence of death, recurrent MI and stent thrombosis [2-4].

In spite of these encouraging results, the heterogeneity of BM cell products and complexity of intercellular interactions in the treated myocardium fuels major controversies in the field. Originally supposed to induce *de novo* cardiomyogenesis [5], BM cells are now mainly acknowledged as promoters of reparative neovascularization [6]. Paracrine communication of transplanted cells with endothelial cells, resident cardiomyocytes and progenitor cells (PCs) recruited from cardiac or distant niches, but also crosstalk between the different cell types within the applied preparation, boosts vascular repair and also conveys survival cues to cardiac cells in the area at risk [6-10]. The extent to which specific BM cell subfractions participate and possibly synergize to determine distinct therapeutic benefits remains a matter of debate [11-13]. Furthermore, risk factors and comorbidities cause pauperization of BM-PCs and shift to the myeloid lineage, together with reduction of regenerative potential and immune competence [8,14-16]. Therefore, transplantation of unselected autologous preparations bears the risk that presence of useless or even harmful cells may hamper the activity of few regenerative cells. In addition, isolation protocols may variably impact on BM cell viability and functionality, thus calling for introduction of quantity and quality control standards [17].

Following these considerations, immunomagnetically and antigenically sorted CD133^+^ or CD34^+^ PCs have been proposed for cardiovascular cell therapy [18-20]. Another attractive option is to select cells on the basis of their functional qualities. This is supported by the observation that the *in vitro* migratory activity towards the chemokine stromal cell-derived factor 1 (SDF-1) predicts the outcome of pre-clinical and human BM cell therapy studies [21,22]. Following this logic, we have developed a cell sorting method based on responsiveness to chemotactic cues. We reported that peripheral blood (PB) MNCs that migrate in response to chemoattractants such as SDF-1 or bradykinin are enriched for CD133^+^ and CD34^+^ PCs, release higher amounts of pro-angiogenic cytokines, and generate more nitric oxide and less superoxide in comparison with non-migrated PB-MNCs [23,24]. Therefore, the *in vitro* migration assay not only recapitulates a fundamental pathophysiological mechanism implicated in tissue repair [25,26], but also provides additional insights into the molecular profile of distinct cell types associated with directed cell motility.

The primary objective of this study was to test if migration towards SDF-1 allows isolating of therapeutically valuable cells from the BM of cardiovascular patients with a history of previous MI. To this end, we compared unfractioned total BM-MNCs and SDF-1-responsive or SDF-1-nonresponsive BM-MNCs with regard to antigenic characteristics, *in vitro* functions, and therapeutic activity in a mouse model of MI. Furthermore, we investigated if differences in secreted pro-angiogenic and pro-survival factors may account for specific therapeutic activities of fractioned cells. In view of clinical translation, BM-MNCs were obtained from coronary artery disease patients undergoing the Bristol-based cell therapy surgical trials TransACT-1 and TransACT-2. Newly results indicate that BM cells from patients with large recent or chronic MI contain populations able to promote cardiac repair by distinct mechanisms involving angiogenesis and cardiomyocyte response to stress.

## Methods

Detailed Methods are provided in Additional file 1.

### Patients

The study was performed in accordance with the declaration of Helsinki (1964) and subsequent amendments and approved by the Bristol National Health Service ethical committee. BM specimens were obtained blindly from risk-profiled coronary artery disease patients participating in the Bristol-based TransACT-1&2 autologous BM cell therapy trials (RA; recruitment completed at the Bristol Heart Institute in January 2014). All necessary informed consent, including acceptance to participate in the study, was obtained from any patients involved in the study. Patients were selected according to strict cardiac magnetic resonance imaging criteria identifying a previous large MI, requiring coronary artery bypass grafting (TransACT-1) or left ventricular restoration surgery (TransACT-2). Key inclusion criteria of the TransACT trials and the anonymized cardiovascular risk profile of the BM donors recruited to this study are reported in Additional file 1: Tables S1 and S2. Details of the clinical trials from which the study is generated are available online [27]. In addition, as a “healthy” reference, BM leftover was obtained from two male individuals (aged 56 and 58 years with no evidence of cardiovascular disease) at the occasion of orthopedic surgery for hip transplantation (covered by National Health Service ethical approval, REC number: 14/WA/1005).

### Isolation of bone marrow mononuclear cell fractions

MNCs were isolated by density centrifugation of fresh human BM cell suspension (5 mM phosphate-buffered saline (PBS)-ethylenediaminetetraacetic acid (EDTA)) on Histopaque 1077 (Sigma, Poole, UK). Migration was performed as previously described using SDF-1 (100 ng/mL) as chemoattractant [23,24]. In some experiments, bradykinin (100 ng/mL) was also used as a stimulus for cell migration. Migration medium without chemoattractant (vehicle) was employed to control for unspecific cell motility. After 18 hours of migration, non-migrating and migrating populations were obtained separately by washing the upper and lower chambers and respective sides of the migration filter three times with 1 × PBS containing 5 mM EDTA. Cell viability was assessed by trypan blue staining. In selected experiments, BM-MNCs were exposed to SDF-1 or vehicle for the same duration of migration.

### Flow cytometry

Unfractioned, non-migrated (SDF-1^non^, veh^non^) and migrated (SDF-1^mig^, veh^mig^) BM-MNCs from 10 individual patients were stained for surface antigen expression and analyzed on a FACS Canto II flow cytometer equipped with FACS Diva software (BD Biosciences, Oxford, UK). Fluorescence minus one staining controls were performed to define positivity.

### Matrigel assay

*In vitro* network formation by human umbilical vein endothelial cells (HUVECs) was studied using a Matrigel assay in the presence of unfractioned or SDF-1^mig^ BM-MNCs. Analysis of cumulative branch length was performed using ImagePro software (Media Cybernetics, Rockville, MD, USA).

### Measurement of growth factors and cytokines

The paracrine activity of unfractioned and SDF-1^mig^ BM-MNCs was quantified by measuring the levels of chemokines (interleukin (IL)-1β, IL-2, IL-3, IL-4, IL-6, IL-7, IL-8, IL-9, IL-10, IL-12, IL-13, and RANTES), chemoattractant factors (granulocyte colony-stimulating factor, granulocyte macrophage colony-stimulating factor, and monocyte chemoattractant protein (MCP-1)), and molecules associated with inflammation and angiogenesis (macrophage inflammatory protein 1α/β, FASL, tumor necrosis factor α, interferon γ, vascular endothelial growth factor (VEGF), basic fibroblast growth factor (bFGF), and angiogenin in cell conditioned media (flow cytometry bead array; BD Biosciences, Oxford, UK). Nine individual samples were studied in duplicate. Furthermore, we used an enzyme-linked immunosorbent assay (ELISA; R&D, Abingdon, UK) for quantitative measurement of human angiogenin in conditioned media (n = 7 patients).

### Angiogenin expression in sorted monocyte subpopulations

BM cells were suspended in PBS-EDTA 5 mM and filtered through a strainer (70 μm sieve). MNCs were isolated by Ficoll Histopaque 1077 (Sigma) gradient. The MNC layer was carefully collected and washed twice in PBS. Cells were stained with PE-conjugated CD3 (MACS Milteny, Bisley, Surrey, United Kingdom), APC-conjugated CD14 (R&D), and PerCP-conjugated CD16 (BioLegend, London, United Kingdom) antibodies for 30 minutes at 4°C. After washing, 5 × 10^7^ MNCs cells were acquired and sorted by Influx™ Cell sorter (BD) into four different groups: CD3^−^CD14^+^CD16^+^, CD3^−^CD14^+^CD16^−^, CD3^−^CD14^−^CD16^+^ and CD3^−^CD14^−^CD16^−^ cells. The expression of angiogenin was then verified in the sorted populations by RT-PCR (n = 4 patients).

### Cell therapy in a murine myocardial infarction model

Animal experiments were performed in accordance with the *Guide for the Care and Use of Laboratory Animals* (Institute of Laboratory Animal Resources, National Academy of Sciences, Bethesda, MD, USA, 1996) and under ethical licence from the UK Home Office (licence holder PM, University of Bristol). MI was induced in 8-week-old immune deficient CD1-FOXN1^nu/nu^ mice (Charles River, Wilmington, MA, US) by ligation of the left anterior descending coronary artery as described previously [28,29]. This was followed by injection of 3 × 10^5^ unfractioned, SDF-1^non^ or SDF-1^mig^ BM-MNCs, or cell-free EBM-2 (vehicle) at three different sites along the infarct border zone with a final volume of 10 μL at each site.

### Echocardiography and hemodynamic measurements

Measurements of dimensional and functional parameters were performed before and 14 days post-MI using a high-frequency, high-resolution echocardiography system (Vevo 770, Visual Sonics, Toronto, Canada) [28,29]. Following the final echocardiography session, intraventricular pressure was measured using a high-fidelity 1.4F transducer tipped catheter (Millar Instruments, Houston, TX, US) inserted into the left ventricle through the right carotid artery. Measurement of echocardiography and pressure indexes was performed in 14 mice per group.

### Immunohistochemistry

Hearts were stopped in diastole by intramyocardial injection of cadmium chloride, washed free of blood by retrograde perfusion with PBS-2% EDTA solution, followed by fixation with 4% paraformaldehyde solution overnight. Cryosections were made at 10 μm thickness, placed on superfrost ultra plus (Thermo, Braunschweig, Germany) slides, air dried for 30 minutes and immunostained for the assessment of capillary and arteriole densities, infiltration of inflammatory cells, interstitial fibrosis and cardiomyocyte proliferation and apoptosis (see Additional file 1).

### Immunocytochemistry of isolated cardiomyocytes

Adult rat cardiomyocytes were isolated using collagenase perfusion and used immediately for experiments. Cells were exposed to hypoxia for 15 hours in cardiomyocyte media mixed with concentrated conditioned media, which were collected from SDF-1^non^ or SDF-1^mig^ or unfractioned BM-MNCs, or EBM-2 basal medium. Synthetic angiogenin (BD, 10 ng/mL) was used as a positive control. In inhibition experiments, recombinant human ribonuclease/angiogenin inhibitor 1 (RNH1; OriGene, Rockville, MD, US) was added to the conditioned medium 1 hour prior to incubation with cardiomyocytes. Cardiomyocytes were fixed and immunostained for the stress granules marker eIF3 (goat polyclonal; Santa Cruz, Dallas, TX, US), α-sarcomeric actinin (mouse monoclonal; Sigma) and 4′,6-diamidino-2-phenylindole to identify nuclei. For cell viability study, cardiomyocytes were incubated for 15 hours in hypoxia with unconditioned medium or conditioned medium from SDF-1^mig^ BM-MNCs, in the presence or absence of RNH1. At the end of the incubation period, rod-shaped cardiomyocytes still attached to the culture-dish were counted as viable cells, while round cells detached from the dish were evaluated as dead cells. Experiments were performed in duplicate. At least 800 cells were analyzed for each condition.

### Statistical analysis

All results are presented as mean ± standard error of the mean, or median and range, from three or more experiments as indicated. For comparison of multiple groups, analysis of variance or Friedman test were used after verification of Gaussian distribution. Statistical significance was then determined by paired or unpaired *t*-tests as appropriate. Furthermore, best-fit and stepwise regression analyses were performed to verify the predictive value of clinical and laboratory data and cell phenotype on outcome endpoints. Variables were entered into the equation only if they satisfied the following criteria: F-to-enter, default value = 3.84; probability of F-to-enter, default value = 0.05. The cumulative effect of these relationships was illustrated by Radar plot. Survival rate was calculated by Kaplan-Meyer test. A *P* value <0.05 was considered significant. Stated n values represent biological replicates.

## Results

### Stromal cell-derived factor 1-induced migration enriches for viable bone marrow mononuclear cells

**Figure 1:**
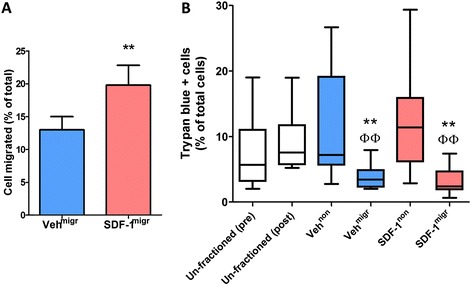
*In vitro* migration allows for separation of viable bone marrow mononuclear cells (BM-MNCs). **(A)** Bar graph shows the percentage of migrated BM-MNCs, following exposure to the chemoattractant stromal cell-derived factor 1 (SDF-1) or vehicle (veh). Values are means ± standard error of the mean from 10 individual donors, with migration (migr) being performed in duplicate. **P < 0.01, versus veh^mig^. **(B)** Box plots show median values, 10th and 90th percentiles, and minimum/maximum values of trypan blue-positive BM-MNCs from the same experiment. Comparison was made between unfractioned total BM-MNCs, either freshly collected (unfractioned pre) or incubated in their original medium for the duration of migration assay (unfractioned post), and BM-MNCs that were harvested from the upper (non) and lower chamber (migr) of the migration assay following stimulation with SDF-1 or its vehicle. Trypan blue-positive BM-MNCs are fewer in the migrated fractions, indicating that spontaneous or directed migration selects for viable cells. **P < 0.001, versus unfractioned BM-MNCs, ^ΦΦ^ *P* < 0.01, versus the respective non-migrated fraction.

Quantitative analysis of migration assays indicates that BM-MNCs from patients with previous large MI are functionally responsive to SDF-1. In fact, the number of BM-MNCs that migrated towards SDF-1 was greater than that of BM-MNCs spontaneously migrating upon exposure to the vehicle (*P* < 0.01; Figure 1A).

Evaluation of viability is a fundamental step in the quality control assessment of cell therapy products*.* An average 5.7% (range 2.0 to 19.1%) of unfractioned BM-MNCs stained positive for trypan blue, a dye that traverses the membrane in a dead cell. This figure did not change after storage of cells in their original medium for the duration of the migration assay (unfractioned post, 7.6%; range 5.2 to 19.0%; Figure 1B). Importantly, trypan blue-positive cells were fewer in the veh^mig^ (3.4%; range 2.0 to 7.9%) and SDF-1^mig^ fractions (2.4%; range 0.7 to 7.4%; *P* < 0.01 versus unfractioned, SDF-1^non^ or veh^non^ BM-MNCs; Figure 1B). A similar migration-induced enrichment in viable cells was obtained using bradykinin as a chemoattractant in the *in vitro* assay (3.0%; range 0 to 7.5%). Altogether, these data indicate that either spontaneous or chemokine-directed migration results in a quality-improved BM cell product.

### Antigenic and molecular characteristics of unfractioned and fractioned bone marrow mononuclear cells

**Figure 2:**
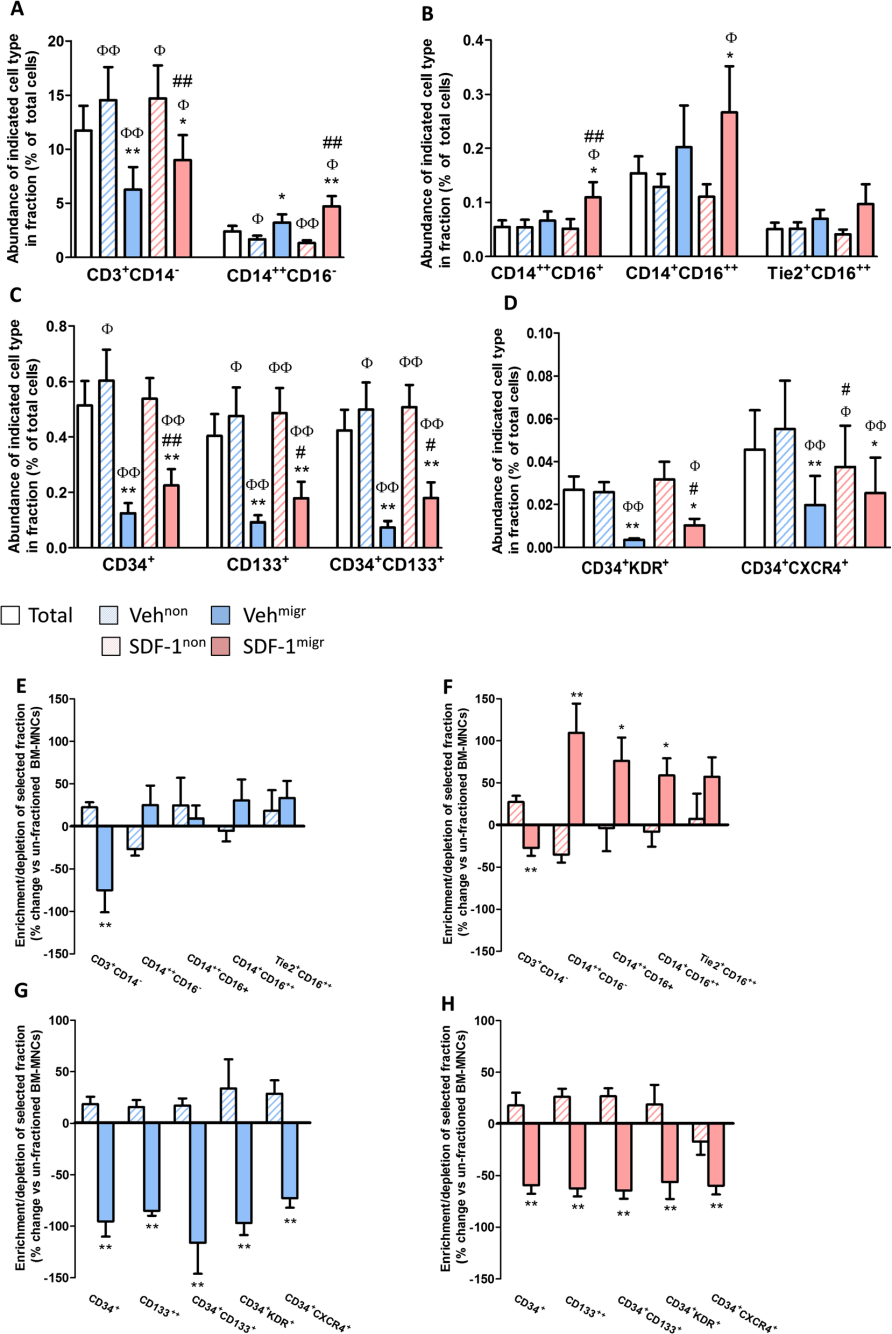
*In vitro* migration leads to enrichment of monocytes and depletion of progenitor cells. **(A-D)** Bar graphs show the percentage of **(A)** lymphocytes and classical monocytes, **(B)** intermediate and non-classical monocytes and **(C,D)** progenitor cells. **(E-H)** Bar graphs show the enrichment/depletion of antigenically defined cell populations in migrated and non-migrated fractions following exposure to **(F,H)** stromal cell-derived factor 1 (SDF-1) or **(E,G)** vehicle (veh). Values are means ± standard error of the mean, n = 10 in each group. ^Φ^ *P* < 0.05, ^ΦΦ^ *P* < 0.01, versus unfractioned; ^#^ *P* < 0.05, ^*##*^ *P* < 0.01, versus corresponding vehicle group; *P < 0.05, **P < 0.01, versus corresponding non-migrated group. BM-MNC, bone marrow mononuclear cell.

We next assessed the cellular composition of unfractioned, migrated and non-migrated populations by flow cytometry. (Gating procedures are shown in Additional file 1: Figure S1.) Granulocytes, which represent approximately 60% of the unfractioned BM population, remained similar in both migrated and non-migrated fractions (data not shown). In contrast, a striking difference between the two fractions was observed with regard to lymphocytes, monocytes, and cells expressing progenitor markers (Figure 2A-D). When considering the percent changes of specific subpopulations before and after migration, we found that spontaneous migration towards vehicle depletes CD3^+^ lymphocytes as well as CD34^+^ and CD133^+^ PCs, co-expressing the VEGF receptor KDR or the SDF-1 receptor CXCR4 (Figure 2E,G). Moreover, SDF-1 stimulation leads to the enrichment of classical (CD14^++^CD16^−^), intermediate (CD14^++^CD16^+^) and non-classical (CD14^+^CD16^++^) monocytes in the migrated fraction (Figure 2F,H). Similar changes in cellular composition were observed when using bradykinin as a chemoattractant in the migration assay (Additional file 1: Figure S2). Regression analysis identified an inverse correlation between the abundance of both CD34^+^ and CD133^+^ PCs in the unfractioned BM-MNC population and patient age (R^2^ = 0.705 and 0.747, respectively; *P* < 0.01). However, we could not find any other associations between cardiovascular risk profile and enrichment/depletion of BM-MNCs following migration.

Verification of the migration assay in BM cells from subjects without a cardiovascular disease background confirms the depletion of CD3^+^ lymphocytes and CD34^+^ and CD133^+^ PCs along with enrichment of CD14^++^CD16^−^, CD14^++^CD16^+^ and CD14^+^CD16^++^ monocytes in the SDF-1-attracted fraction (Additional file 1: Figure S3).

### Support of endothelial tube formation and paracrine factors secreted by unfractioned and fractioned bone marrow mononuclear cells

**Figure 3:**
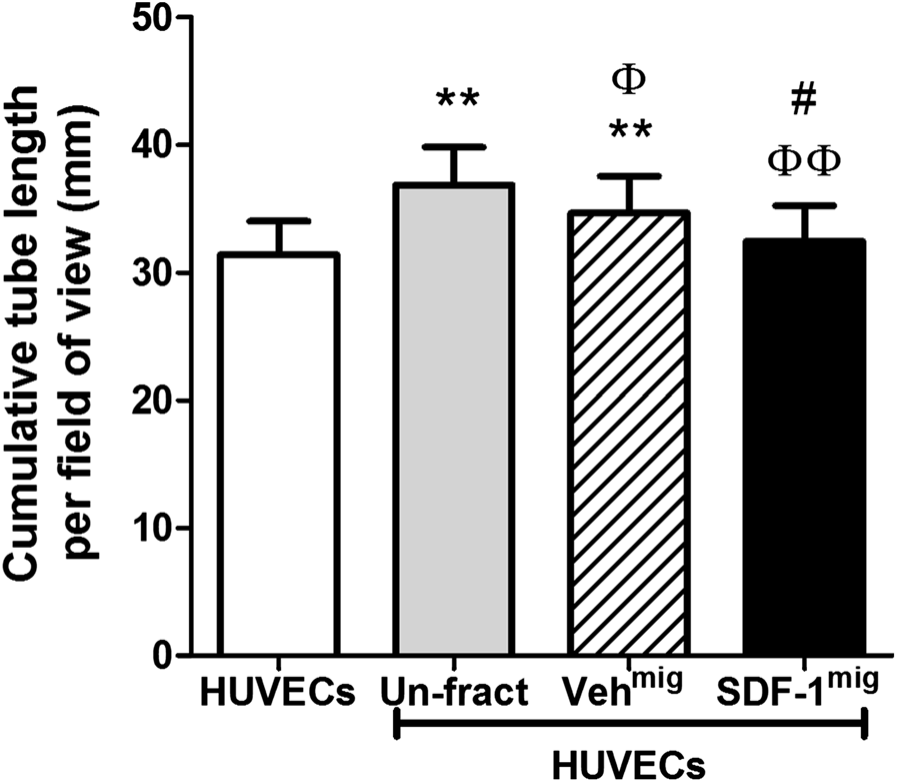
Comparison of *in vitro* endothelial network formation potentiation by unfractioned bone marrow mononuclear cells (BM-MNCs). Bar graph shows that unfractioned BM-MNCs support *in vitro* network formation by human umbilical vein endothelial cells (HUVECs) more efficiently than stromal cell-derived factor 1 migrated (SDF-1^mig^) counterparts. BM-MNCs were obtained from 10 donors for matched comparisons. Values are means ± standard error of the mean. **P < 0.01, versus HUVECs; ^Φ^ *P* < 0.05, ^ΦΦ^ *P* < 0.01, versus unfractioned BM-MNCs; ^*#*^ *P* < 0.05, versus vehicle migrated (Veh^mig^).

We next investigated if differences in cellular composition before and after migration are associated with changes in pro-angiogenic activity on Matrigel. Unfractioned BM-MNCs augmented the network forming capacity of HUVECs (*P* < 0.01 versus HUVECs alone; Figure 3). Regression analysis indicates that the *in vitro* pro-angiogenic activity of unfractioned BM-MNCs is predicted by the relative abundance of CD34^+^ and CD133^+^ PCs (*P* < 0.02 for both cells types) and CD14^++^CD16^−^ (*P* < 0.01) and Tie2^+^CD16^+^ monocytes (*P* < 0.05). In contrast, SDF-1^mig^ cells, which are enriched in monocytes but depleted of CD34^+^ and CD133^+^ PCs, did not support endothelial network formation (*P* < 0.01 versus unfractioned, not significant versus HUVECs alone; Figure 3). These data suggest that the cooperation of progenitor populations with other cell subfractions is essential for promotion of *in vitro* network formation by HUVECs.

BM-MNCs secrete growth factors and cytokines that influence angiogenesis *in vitro* and *in vivo* [10]. Using a cytometric bead array that measures a variety of cytokines and growth factors implicated in reparative angiogenesis, we found that SDF-1^mig^ BM-MNCs release angiogenin more abundantly than unfractioned BM-MNCs (*P* < 0.01), while the latter tend to release more VEGF, tumor necrosis factor-α and RANTES (Additional file 1: Figure S4A). Regression analysis revealed that the association of different secreted factors (for example, VEGF, IL-8, angiogenin and MCP-1) predicts the increase in endothelial network formation induced by unfractioned BM-MNCs (*P* < 0.01). This cooperative interaction is also illustrated by the radar plot generated by connecting the F-to-enter values from regression analyses (Additional file 1: Figure S4B).

**Figure 4:**
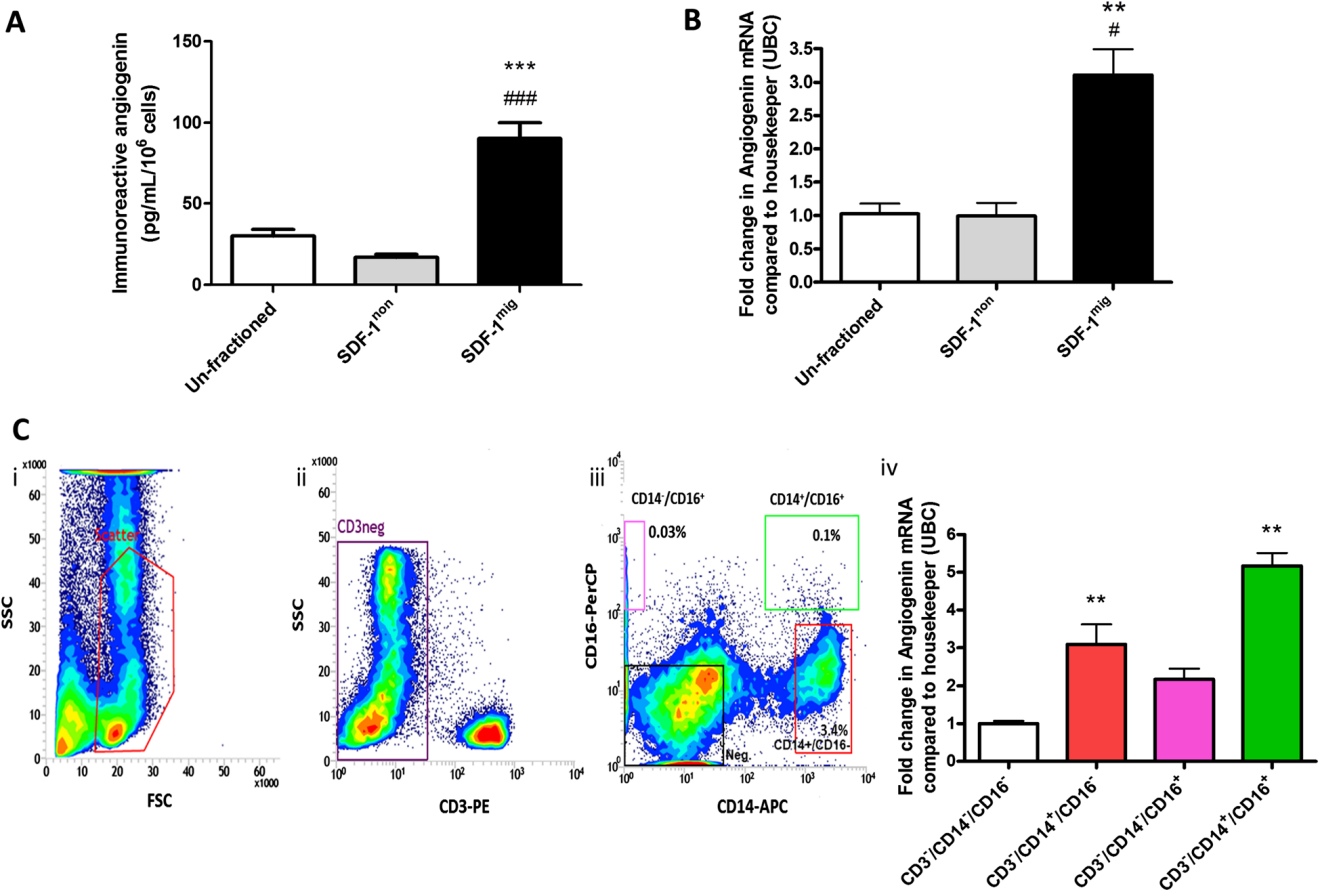
Expression of angiogenin in migrated and sorted bone marrow cell populations. **(A)** Immunoreactive angiogenin levels in conditioned media from unfractioned, stromal cell-derived factor 1 non migrated (SDF-1^non^) and migrated (SDF-1^mig^) mononuclear cells (MNCs; n = 7 per group). **(B)** Angiogenin mRNA levels of unfractioned, SDF-1^non^ and SDF-1^mig^ MNCs (n = 4 per group). Values are means ± standard error of the mean. *P < 0.05, **P < 0.01, ***P < 0.001, versus unfractioned; ^#^ *P* < 0.05, ^###^ *P* < 0.001, versus SDF-1^non^. **(C)** Angiogenin mRNA levels in sorted bone marrow cells. (i-iii**)** Gating strategy to sort BM monocyte subpopulations: human MNCs isolated by Ficoll gradient from BM were analyzed by flow cytometry (i). Lympho-monocyte population was gated and analyzed according to CD3 positivity (ii) in order to exclude lymphocytes. Cells negative for CD3 were gated and further analyzed for monocytes markers CD14 and CD16 (iii). Four distinct populations were sorted: CD14^−^/CD16^+^ (pink gate), CD14^+^/CD16^+^ (green gate), CD14^+^/CD16^−^ (red gate), negative cells (black gate). Angiogenin levels were measured in triplicate by RT-PCR (iv). Values are means ± standard error of the mean. **P < 0.01, versus CD3^−^/CD14^−^/CD16^−^ cells. FSC, forward-scattered light; SSC, side-scattered light; UBC, Ubiquitin C.

In order to verify the levels of angiogenin with a more sensitive method, we assayed the factor by a specific ELISA. The assay confirmed higher levels of angiogenin in conditioned media of SDF-1^mig^ cells (90 ± 10 pg/mL per million cells) as compared with the respective unfractioned (30 ± 4; *P* < 0.001) and SDF-1^non^ cells (17 ± 2; *P* < 0.001; n = 7 donors; Figure 4A). RT-PCR verified that SDF-1^mig^ cells express more angiogenin that unfractioned (3.2-fold) and SDF-1^non^ cells (4.7-fold) (*P* < 0.05 and *P* < 0.01, respectively; n = 4 donors; Figure 4B). In contrast, no change in angiogenin protein and mRNA levels was observed when exposing unfractioned BM-MNCs to SDF-1 in a test tube for the duration of the migration assay, thus suggesting that directed migration to, rather than SDF-1 itself, is responsible for the observed difference in angiogenin expression (data not shown). In order to identify the BM-MNC fraction that expresses angiogenin, we sorted total BM cells according to the gating procedure illustrated in Figure 4C. We found that CD14^+^CD16^+^ and CD14^+^CD16^−^ monocytes express five-fold and three-fold higher angiogenin levels as compared to non-monocyte populations. These data newly identify angiogenin-expressing monocytes in the migratory element of BM obtained from MI patients.

### *In vivo* therapeutic activity of unfractioned and migration-fractioned bone marrow mononuclear cells

We next asked if BM cell populations selected by SDF-1-induced migration differ from total BM cells with regard to therapeutic activity in ischemia. To this end, we injected SDF-1^non^, SDF-1^mig^ or unfractioned BM-MNCs from three donors into the infarct border zone of immunodeficient mice.

**Figure 5:**
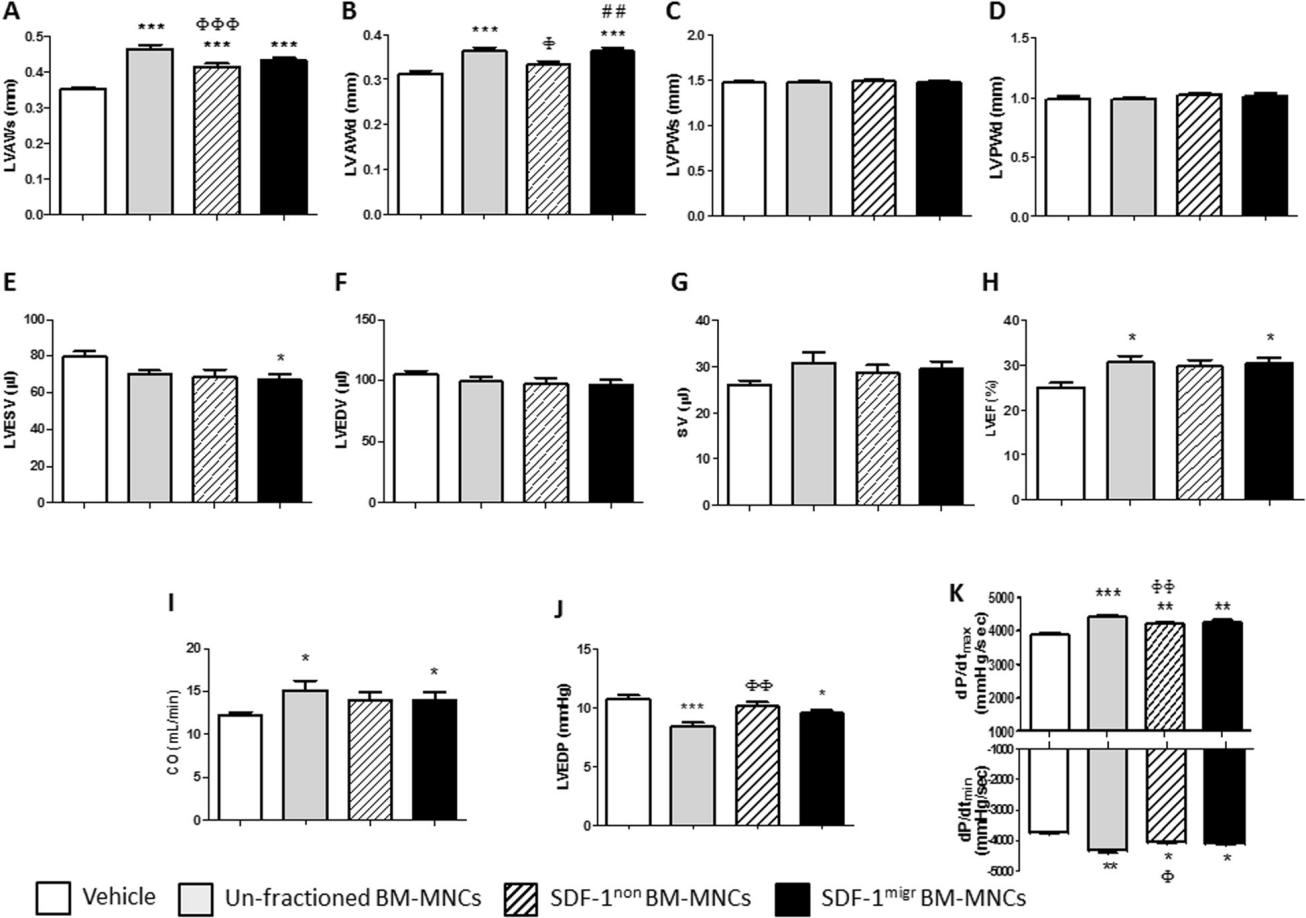
Intra-myocardial injection of unfractioned and migration-enriched bone marrow mononuclear cells (BM-MNCs) improves echocardiography endpoints in a immune-deficient mouse model of myocardial infarction. Bar graphs show the effect of unfractioned, stromal cell-derived factor 1 (SDF-1) migrated (migr) or non-migrated (non) BM-MNCs from three different donors on **(A-I)** indexes of contractility and **(J,K)** intraventricular pressure in immunodeficient mice with myocardial infarction. N = 14 mice per group for all the measured indexes. *P < 0.05, **P < 0.01, ***P < 0.001, versus vehicle; ^Φ^ *P* < 0.05, ^ΦΦ^ *P* < 0.01, ^ΦΦΦ^ *P* < 0.001, versus unfractioned BM-MNCs; ^##^ *P* < 0.01, versus non-migrated BM-MNCs. CO, cardiac output; dP/dt, maximum changes in developed pressure; LVAWd, left ventricular anterior wall thickness in diastole; LVAWs, left ventricular anterior wall thickness in systole; LVEDP, left ventricular end diastolic pressure; LVEDV, left ventricular end diastolic volume; LVEF, left ventricular ejection fraction; LVESV, left ventricular end systolic volume; LVPWd, left ventricular posterior wall thickness in diastole; LVPWs, left ventricular posterior wall thickness in systole; SV, stroke volume.

As shown in Additional file 1 (Figure S5), cell therapy improves post-MI survival rate as compared with vehicle (*P* < 0.02), with no difference between migrated, non-migrated or unfractioned BM-MNCs. No differences were detected for echocardiographic parameters between recipient groups at day 0 (data not shown). Analysis of variance detected an effect of cell therapy with regard to left ventricular anterior wall thickness (*P* < 0.0001), left ventricular end systolic volume (*P* < 0.05), left ventricular ejection fraction (LVEF; *P* = 0.01), and cardiac output (*P* < 0.05) (Figure 5A-I), with no difference among donors. Comparison of the effects induced by distinct BM-MNC fractions by Tukey’s test indicates that unfractioned and SDF-1^migr^ BM-MNCs induce comparable improvements in left ventricular anterior wall thickness (*P* < 0.001 versus vehicle for both comparisons; Figure 5A,B), LVEF (*P* < 0.05 versus vehicle for both comparisons; Figure 5H), and cardiac output (*P* < 0.05 versus vehicle for both comparisons; Figure 5I). In contrast, there was no difference between groups that received SDF-1^non^ cells or vehicle with regard to indexes of contractility (that is, LVEF and cardiac output).

Cell therapy improved left ventricular end diastolic pressure, and maximum and minimum changes in developed pressure (*P* < 0.0001 versus vehicle; Figure 5J,K), with no difference among donors. When examining the effects of distinct cell fractions, we found that injections of unfractioned or SDF-1^migr^ BM-MNCs blunts the post-MI increase in left ventricular end diastolic pressure (*P* < 0.001 and *P* < 0.05 versus vehicle, respectively), while SDF-1^non^ BM-MNCs were ineffective (not significant versus vehicle, *P* < 0.01 versus unfractioned BM-MNCs; Figure 5J). Likewise, SDF-1^non^ BM-MNCs were inferior to total BM-MNCs in preserving the maximum and minimum changes in developed pressure indexes (Figure 5K). Taken together, these data indicate that injections of unfractioned or migrated BM-MNCs improves left ventricular remodeling and contractility to a greater extent than the non-migrated counterparts.

**Figure 6:**
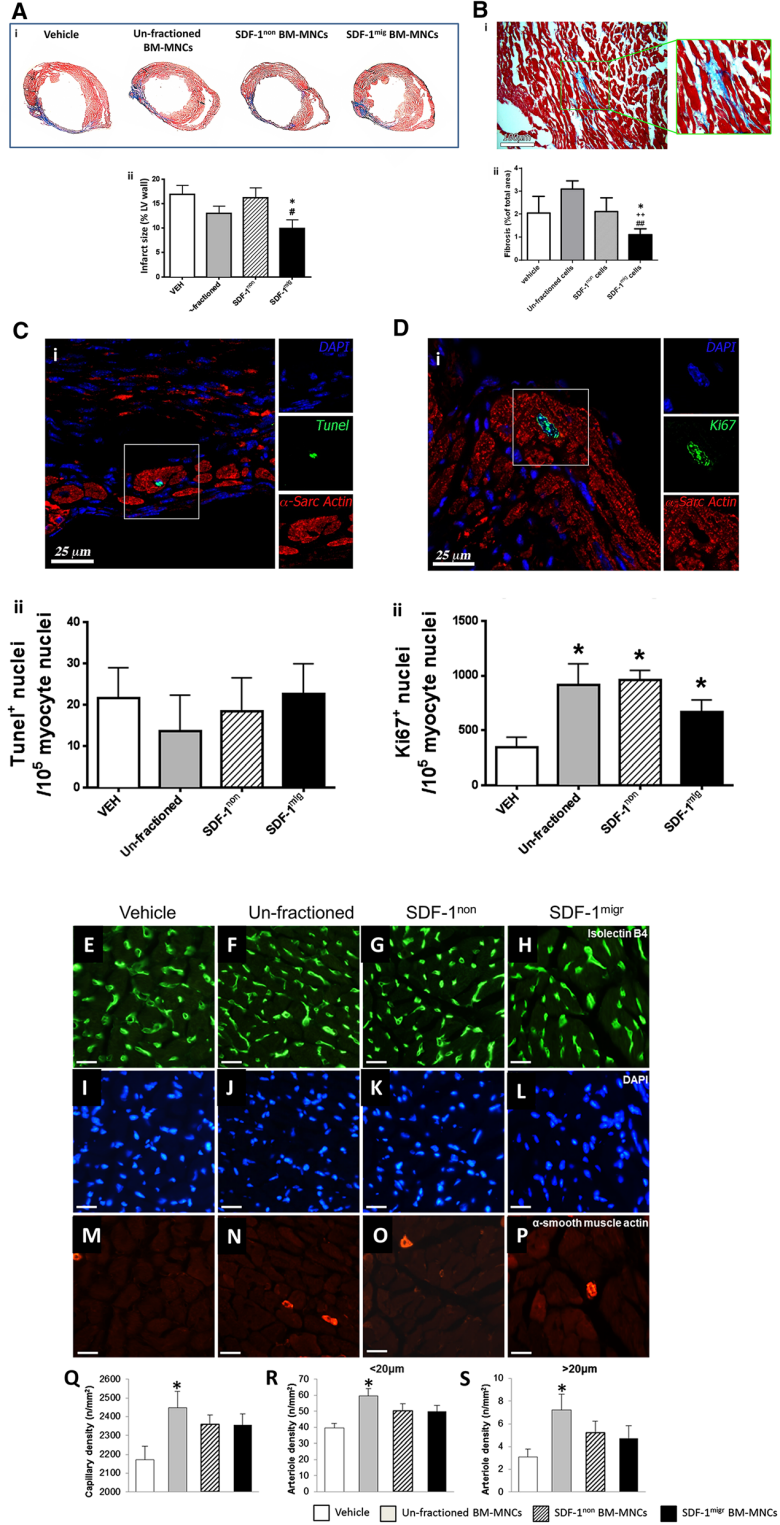
Effect of cell therapy on infarct size, interstitial fibrosis and peri-infarct vascularization. **(A)** Infarct size assessed at 2 weeks after myocardial infaction induction and injection of bone marrow mononuclear cells (BM-MNCs) or vehicle (VEH). (i) Representative images and (ii) bar graph showing average values. **(B)** Peri-infarct interstitial fibrosis. (i) Representative image and (ii) bar graph showing average values. In the representative image and insert magnification, cardiomyocytes are stained red, while the fibrotic tissue is stained blue. **(C,D)** Fraction of Tunel positive **(C)** and KI-67 positive **(D)** cardiomyocytes. Representative images of immunostaining (i) and average values (ii). **(E-S)** Myocardial vascularization assessed by counting the density of capillaries and arterioles in the LV peri-infarct zone. Representative immunohistochemistry images **(E-P)** and average values **(Q-S)**. White scale bar = 20 μm. N = 7 to 10 per group. Values are means ± standard error of the mean. *P < 0.05, versus vehicle; ^++^ *P* < 0.01, versus unfractioned; ^#^ *P* < 0.05; ^##^ *P* < 0.01, versus SDF-1^non^. LV, left ventricular; SDF-1^mig^, stromal cell-derived factor 1 migrated; SDF-1^non^, stromal cell-derived factor 1 non-migrated.

We next verified if the denoted hemodynamic improvement is associated with a reduction of the scar and increase in reparative neovascularization. Masson trichrome staining showed a reduction in infarct scar and peri-infarct interstitial fibrosis in the group that received SDF-1^migr^ BM-MNCs, but not in the other cell therapy groups (Figure 6A,B). Immunohistochemistry analyses denoted no group difference with regard to the abundance of apoptotic cardiomyocytes, but an overall increase in Ki-67-positive cardiomyocytes in the peri-infarct zone of the cell therapy groups compared with vehicle (Figure 6C,D).

Analysis of the coronary microvasculature showed that mice receiving cell therapy exhibit higher levels of capillaries and arterioles than mice given vehicle (*P* < 0.01; Figure 6E-S). *Post-hoc* analysis by Tukey’s test revealed the superior pro-angiogenic activity of unfractioned BM-MNCs versus SDF-1^non^ or SDF-1^mig^ cells, in line with results of *in vitro* network formation on Matrigel. Histological studies also revealed an effect of cell therapy on the number of infiltrating CD45^+^ cells (unfractioned, 21 ± 2; SDF-1^non^, 28 ± 2; SDF-1^mig^, 25 ± 2; versus vehicle, 52 ± 4 cells/mm^2^; *P* < 0.01 for all comparisons), with no difference between cell therapy groups.

Altogether, these data indicate that unfractioned and SDF-1^migr^ BM-MNCs exert similar therapeutic effects as assessed by echocardiography and intraventricular pressure measurements. However, only unfractioned cells have pro-angiogenic activity, whereas SDF-1^migr^ cells reduce infarct size and interstitial fibrosis.

### Stromal cell-derived factor 1 migrated bone marrow mononuclear cells induce the formation of stress granules in hypoxic cardiomyocytes through an angiogenin-mediated mechanism

**Figure 7:**
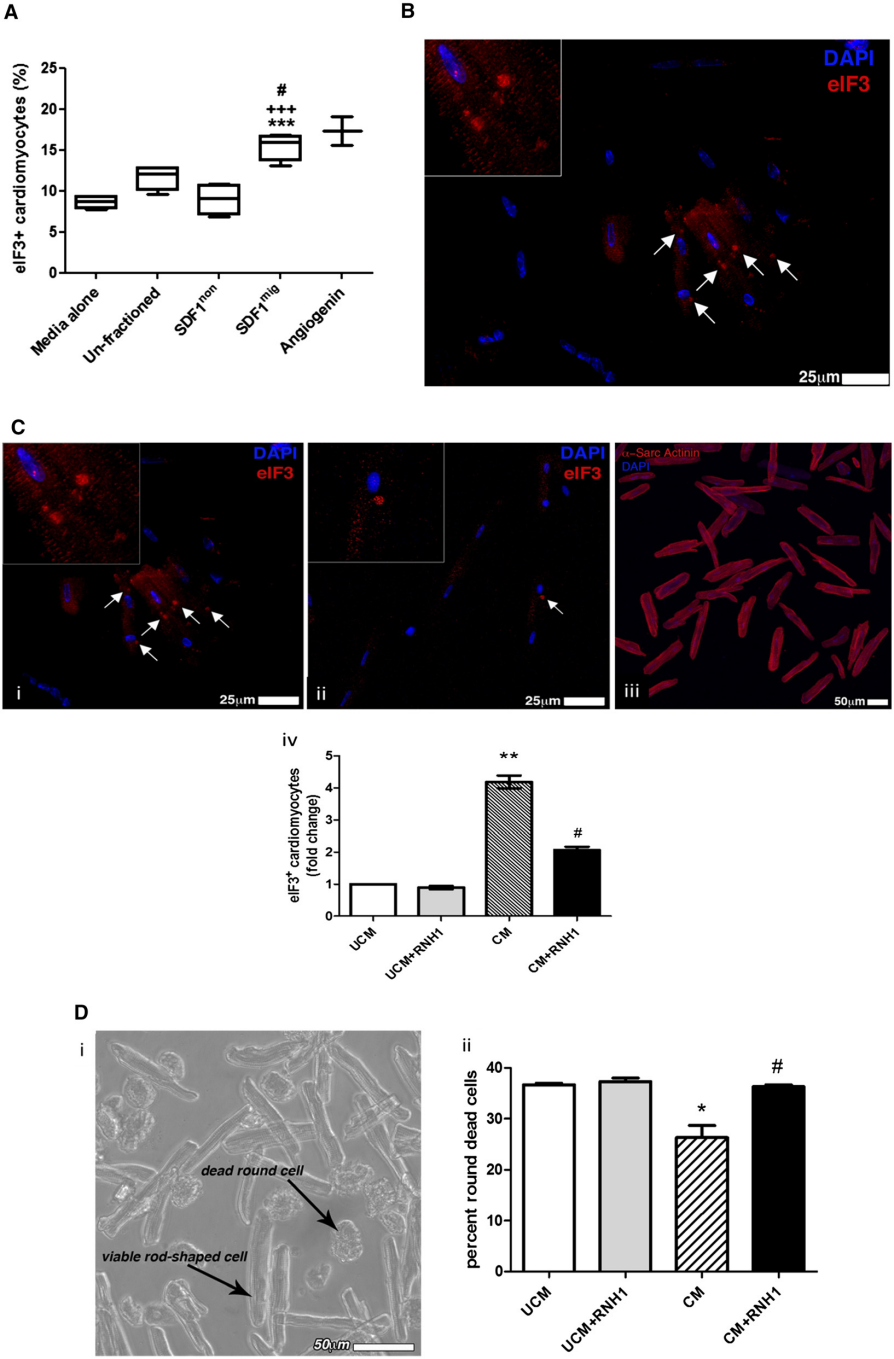
Formation of stress granules (SGs) in hypoxic cardiomyocytes following stimulation with bone marrow mononuclear cell (BM-MNC)-conditioned medium. **(A)** Box plots show median values, 10th and 90th percentiles, and minimum/maximum values of hypoxic cardiomyocytes that express eIF3-positive SGs following stimulation with unconditioned medium or conditioned media from unfractioned, SDF-1^non^ or SDF-1-^mig^ BM-MNCs. Human angiogenin was used as a positive control. Values are means ± standard error of the mean. ***P < 0.001, versus unconditioned medium; ^#^ *P* < 0.001, versus unfractioned; ^+++^ *P* < 0.001, versus SDF-1^non^. **(B)** Representative fluorescent microscopy image of SGs. **(C)** Inhibition of angiogenin by ribonuclease/angiogenin inhibitor 1 (RNH1) results in a reduction in the ability of SDF-1^mig^ cell-conditioned medium to stimulate the formation of SGs. Representative images of eIF3 granules in cardiomyocytes exposed to SDF-1^mig^ BM-MNC-conditioned medium in the absence (i) or presence (ii) of RNH1 inhibitor. Myocyte cytoplasm is labeled with alpha sarcomeric actinin (iii). Nuclei are depicted in blue (4′,6-diamidino-2-phenylindole; DAPI). (iv) Graph showing average results. **P < 0.05, versus unconditioned medium (UCM); ^**#**^ *P* < 0.05, versus of SDF-1^mig^ cell-conditioned medium (CM). **(D)** Inhibition of angiogenin by RNH1 results in a reduction in the ability of SDF-1^mig^ cell-conditioned medium to contrast cardiomyocyte death upon exposure to hypoxia. Round-shaped cells indicate dead cells, while rod-shaped cells are viable cells. *P < 0.05, versus UCM; ^**#**^ *P* < 0.05, versus of SDF-1^mig^ cell CM. SDF-1^mig^, stromal cell-derived factor 1 migrated; SDF-1^non^, stromal cell-derived factor 1 non-migrated.

Recent studies indicate that, under stress conditions, angiogenin enzymatically generates tRNA-derived stress-induced small RNA (tiRNA) in cytoplasmic stress granules (SGs), thereby restricting translation to the synthesis of proteins necessary for cell survival [30]. Here, we investigated if BM-MNC-secreted angiogenin may influence cardiomyocyte viability. Immunocytochemistry analyses of rat cardiomyocytes exposed to hypoxia in the presence of BM-MNC-conditioned media showed an increase in the percentage of cells expressing eIF3 positive SGs (analysis of variance, *P* < 0.001 versus unconditioned medium; Figure 7A,B). Tukey’s multiple comparison test indicates an increase of SGs in cardiomyocytes exposed to the SDF-1^mig^ cell-conditioned medium compared to cardiomyocytes exposed to SDF-1^non^ (*P* < 0.01) or unfractioned cell-conditioned media (*P* < 0.05). The effect induced by SDF-1^mig^ cells was similar to that observed by incubating cardiomyocytes with synthetic angiogenin (positive control).

Importantly, the induction of SGs by SDF-1^mig^ cell-conditioned medium (*P* < 0.05 versus unconditioned medium) was reduced by co-incubation with RNH1, a ribonuclease/angiogenin inhibitor (*P* < 0.05 versus conditioned medium), thus indicating the involvement of secreted angiogenin in the augmentation of cardiomyocyte stress response (Figure 7C). Additionally, SDF-1^mig^ cell-conditioned medium increases the viability of hypoxic cardiomyocytes, with this effect being reduced by RNH1 (Figure 7D). Altogether, these data indicate a novel cardioprotective effect of migrating BM cells, involving angiogenin-mediated stress response in hypoxic cardiomyocytes.

## Discussion

Results of the present study indicate that migration-based selection separates two distinct fractions from total BM cells of patients with previous MI: the non-migrated component contains an abundance of lymphocytes and CD34^+^/CD133^+^ PCs, while the migrated element is more enriched with classical, intermediate and non-classical monocytes. Furthermore, intramyocardial injection of unfractioned or migrated cells induces similar therapeutic benefits in a murine model of MI, but through different mechanisms. As expected, unfractioned cells promote reparative angiogenesis; in contrast, we show for the first time that SDF-1^migr^ cells reduce the infarct size and peri-infarct fibrosis and exert cardiac protection through the release of angiogenin and potentiation of stress response in hypoxic cardiomyocytes. The present study was limited to 14 days post-MI, as the *a priori* endpoint was to discriminate differences in early reparative responses to BM cell therapy that can be revealed by the migration-based fractioning. More investigation is necessary to determine the impact of the fractioning approach on prevention of late cardiac remodeling and heart failure.

Advances in clinical management over the last decades have led to a marked improvement in the survival rate of patients suffering from acute MI. However, due to the permanent loss of viable myocardium, a large proportion of survivors develop cardiac dysfunction, which calls for novel regenerative treatments. To date, the most successful clinical trials performing autologous BM-MNC transplantation in MI patients achieved an improvement of LVEF by up to 20% and a decrease in infarct size of up to 30% [31-36]. However, when looking at meta-analysis reports, the size of the beneficial effects has varied grossly, often due to methodological differences. Several studies have since attempted further refinement by modulating the composition of the transplanted cells with the aim of selecting for a presumed most efficient cell type [37-41]. However, efforts have been often hindered by functional limitations and weakened regenerative capacity intrinsic to patient-derived cells [15,42-44]. In addition, cell damage can occur as result of harvesting procedures and subsequent processing prior to transplantation. The novel observation that migration fractioning significantly increases the number of viable cells from the total BM preparation and enriches for cells endowed with specific reparative activity has therefore, in our opinion, a relevant translational value.

Cardiovascular risk factors and ageing induce quantitative and functional defects in BM PCs. Consistently, in the present study, the number of CD34^+^ PCs was influenced by patient’s age, (that is, older patients had lower CD34^+^ cell counts in total BM-MNCs). Another study showed that age influences the number and colony-forming capacity of circulating CD34^+^ PCs [45]. Furthermore, we have previously observed that migration towards bradykinin enriches for pro-angiogenic PCs from PB-MNCs of healthy donors. However, migration-based enrichment for PCs was significantly blunted, although not completed abrogated, in acute MI patients. In the present study, conducted in patients with previous MI, SDF-1-induced migration resulted in the depletion of BM CD34^+^ and CD133^+^ PCs. The discrepant behavior of BM- and PB-derived PCs is intriguing and may reflect a “migratory immaturity” of BM-resident PCs, as opposed to circulating PCs which are already primed to mobilization. Impaired CD34^+^ PC migration is reportedly associated with adverse events in patients with MI. The migratory defect might be attributable to the downregulation of receptor expression or the dysfunction of signaling pathways downstream of the receptor, as it has been reported for circulating cells before [46-48]. In addition, increased cleavage of SDF-1 by circulating or cell membrane-bound proteases, like dipeptidylpeptidase IV/CD26, might result in the loss of its signaling capabilities [49]. Other possibilities include the choice of stimulus and the time period allowed for migration. When testing those hypotheses, we did not observe any difference using bradykinin instead of SDF-1. Likewise, similar results were obtained in experiments where migration was performed for 6 or 18 hours (data not shown).

Of note, we show that different cell populations within the total BM-MNC fraction contribute together in stimulation of angiogenesis. Separation of PC and monocyte components by migration resulted in the loss of pro-angiogenic activity. These findings might have high translational value for cell therapy applications, in particular with respect to the choice of delivering BM cell products directly or systemically [50]. They imply that BM-derived cells from MI patients might not be able to provide their key pro-angiogenic effect in the MI area if delivered through a route (like the intracoronary delivery approach, for example) that relies on cell intrinsic migration potential to reach the MI area. We additionally found that lymphocytes are predominantly retained in the non-migrated fraction. There is a lack of consensus on the role played by lymphocytes in reparative processes, as these cells have been reported either to inhibit [51] or promote angiogenesis [52], including by shedding of microvesicles, which in turn can stimulate vascular growth [53]. However, the present study did not show any association between lymphocytes and promotion of *in vitro* angiogenesis by unfractioned BM cells.

Different secreted factors, including VEGF, IL-8, MCP-1 and angiogenin, were complementary in stimulation of *in vitro* angiogenesis by total BM cells. Surprisingly, however, transplantation of unfractioned BM-MNCs or SDF-1^mig^ BM-MNCs, which are enriched with angiogenin-expressing monocytes, promoted similar functional recovery in a mouse MI model, but only the former improved reparative angiogenesis *in vivo*. On the other hand, SDF-1^mig^ BM cells induced a significant reduction of the infarct size and interstitial fibrosis, while unfractioned BM-MNCs did not. Interestingly, a recent pre-clinical cell therapy study in rats with acute MI using mesenchymal stem cells (MSCs) transfected with an adenoviral vector carrying the angiogenin gene (MSC^AdANG^) showed that angiogenin expression confers donor cells with high resistance to oxygen deprivation and with increased capacity to reduce infarct size, left ventricular remodeling and improve cardiac function [54]. At variance with our study, cell therapy with MSC^AdANG^ also induced reparative angiogenesis in the peri-infarct zone [54]. However, it should be noted that MSC^AdANG^ secrete much higher levels of angiogenin (~700 ng/mL) as compared with SDF-1^mig^ BM-MNCs (~100 pg/mL). The activity of angiogenin is relatively low compared with that of other angiogenic factors. Therefore, differences in angiogenin dosage may account for the distinction between MSC^AdANG^ and SDF-1^mig^ BM-MNCs with regard to promotion of reparative angiogenesis.

Emerging evidence indicates that, besides inducing the transcription of various angiogenic genes [30], angiogenin promotes stress responses instrumental to cell survival under adverse conditions [55]. In particular, angiogenin acts as an RNAase cleaving tRNAs into stress-induced tiRNAs, which in turn suppress the synthesis of housekeeping proteins by inhibiting mRNA translation [56]. Translationally repressed messenger ribonucleoproteins are then transiently stored in SGs, which are dynamic structures that can be recycled as cells recover from stress [57]. Importantly, translation of IRES-dependent mRNAs, many of which are involved in pro-survival and anti-apoptosis, is resistant to tiRNA-dependent inhibition, thereby allowing stressed cells to direct the translational machinery and residual energy to functions instrumental to survival [58,59]. Additional pro-survival mechanisms of angiogenin include an interaction with the p53, p21 and Bax pathway in cancer cells [60]. In line with results on other cell types [57,61], we report for the first time that exogenous angiogenin stimulates the formation of SGs in isolated rat cardiomyocytes. Most importantly, we newly show that SDF-1^mig^ BM-MNCs express and release large amounts of angiogenin and that the conditioned medium of these cells enhances the formation of SGs in rat cardiomyocytes exposed to hypoxia. This was associated with a reduction of cardiomyocyte death. An implication of angiogenin in the formation of SGs in cardiomyocytes exposed to SDF-1^migr^ cell media is confirmed by the finding that this effect is abrogated by an angiogenin inhibitor. Similarly inhibited was the improvement in cardiomyocyte survival. These new data highlight a novel mechanism that may contribute to cardiac protection elicited by the migrating component of BM-MNCs. It should be noted that, in our *in vivo* study, cell therapy did not produce any significant reduction in cardiomyocyte apoptosis at the level of the peri-infarct zone, as detected by Tunel staining at 2 weeks post-MI. Cardiomyocyte loss is prevalent in the early phase after permanent coronary artery ligation and therefore any benefit induced by cell therapy might have been underestimated in our study. Furthermore, it is not clear whether angiogenin-induced protection derives from inhibition of “programmed, apoptotic” or “accidental, non-apoptotic” cell death. More in-depth investigation is warranted to address this aspect.

Sorting experiments newly show that, within BM-MNCs, CD14^+^CD16^+^ monocytes express the highest levels of angiogenin. Experimental evidence indicates that healing of infarcted heart requires monocytes/macrophages. Classical monocytes, recruited in the early phase after an MI, degrade cellular debris and macromolecules at the infarct site, while non-classical monocytes intervene later, modulating the formation of granulation tissue and cardiac remodeling [8]. Previous studies by Nahrendorf and colleagues have shown that Ly-6Clo (mouse analogues of CD14^+^CD16^+^ monocytes) promote myocardial healing via myofibroblast accumulation, angiogenesis, and deposition of collagen [8]. Although circulating levels did not change following MI induction, the capacity of Ly-6Clo/CD14^+^CD16^+^ monocytes to migrate into ischemic myocardium increased 4.8-fold. This unique homing capacity has been attributed to the expression of different chemokine receptors, including CX3CR1, CCR5 and the SDF-1 receptor CXCR4. Due to their ability to sense migratory cues, angiogenin-expressing monocytes could spread through the peri-infarct area and exert protective effect on stunned cardiomyocytes. Therefore, the expression of chemokine receptors and pro-survival factors may represent a fundamental association for monocyte healing capacity.

## Conclusions

This study conducted on BM specimens of TransACT cell therapy trials newly demonstrates that migratory features of BM-MNCs are associated with distinct cardioprotective modalities. These new findings improve current understanding of mechanisms implicated in spontaneous and cell therapy-induced cardiac repair. On the other hand, this research was not designed to make any assumption or prediction of the trial outcomes. The TransAct studies compare the benefit of intramyocardial delivery of BM CD133^+^ PCs or their medium, in combination with surgical revascularization or ventricular reshaping in patients with previous MI, whereas here we verified the therapeutic effect of BM subfractions in a mouse model of acute MI induced by permanent coronary artery ligation. While additional investigation is necessary to confirm the actions of migrated and non-migrated fractions in models of ischemia-reperfusion or chronic ischemia, this study highlights the mechanistic complexity of current cell therapy and the requirement of standardized cell products in future clinical trials.

## Additional file

Additional file 1: Table S1. Key inclusion criteria of TransACT trials. **Table S2.** Basic cardiovascular risk profile of BM donors. **Figure S1.** Gating strategy for antigenic characterization of BM-MNCs by flow cytometry. **Figure S2.** *In vitro* migration towards bradykinin alters the composition of BM-MNCs. **Figure S3.** *In vitro* migration of BM-MNCs from subjects without cardiovascular disease. **Figure S4.** Paracrine activity of BM-MNCs. **Figure S5.** Shows overall survival in MI mice intramyocardially injected with unfractioned BM-MNCs, migrated (SDF-1^migr^) or non-migrated (SDF-1^non^) fractions.

## Abbreviations

BM: bone marrow
BM-MNC: bone marrow mononuclear cell
EDTA: ethylenediaminetetraacetic acid
ELISA: enzyme-linked immunosorbent assay
HUVEC: human umbilical vein endothelial cell
IL: interleukin
LVEF: left ventricular ejection fraction
MCP: monocyte chemoattractant protein
MI: myocardial infarction
MSC: mesenchymal stem cell
PB: peripheral blood
PBS: phosphate-buffered saline
PC: progenitor cell
RNH1: ribonuclease/angiogenin inhibitor 1
SDF-1: stromal cell-derived factor 1
SDF-1^mig^: SDF-1 migrated
SDF-1^non^: SDF-1 non-migrated
SG: stress granule
tiRNA: stress-induced small RNA
VEGF: vascular endothelial growth factor
Veh^mig^: vehicle migrated
Veh^non^: vehicle non-migrated
