# Supplementary material for: Migration towards SDF-1 selects angiogenin-expressing bone marrow monocytes endowed with cardiac reparative activity in patients with previous myocardial infarction
Source: Stem Cell Res Ther. 2015 Apr 11;6(1):53. doi: 10.1186/s13287-015-0028-y (PMC4440500; doi:10.1186/s13287-015-0028-y)
Supplement: Additional file 1: Table S1. — Key inclusion criteria of TransACT trials. Table S2. Basic cardiovascular risk profile of BM donors. Figure S1. Gating strategy for antigenic characterization of BM-MNCs by flow cytometry. Figure S2. In vitro migration towards bradykinin alters the composition of BM-MNCs. Figure S3. In vitro migration of BM-MNCs from subjects without cardiovascular disease. Figure S4. Paracrine activity of BM-MNCs. Figure S5. Shows overall survival in MI mice intramyocardially injected with unfractioned BM-MNCs, migrated (SDF-1migr) or non-migrated (SDF-1non) fractions. [file 13287_2015_28_MOESM1_ESM.docx]

### SUPPLEMENTARY METHODS

**In vivo experimental procedures**

The study was performed in accordance with the Guide for the Care and Use of Laboratory Animals (Institute of Laboratory Animal Resources, 1996) and with approval of the British Home Office and the University of Bristol.

**Cardiomyocyte experiments**

Adult male rats were killed by stunning and cervical dislocation prior to dissection of the heart. Ventricular cardiomyocytes were isolated by the Langendorff method, using collagenase perfusion. At the end of the isolation procedure, Calcium was reintroduced gradually in the cell suspension to reach a concentration of 1.8 mM. Cardiomyocytes were then plated at a density of 10,000 cells/cm^2^ on murine laminin (Sigma-Aldrich, 5μg/cm^2^) coated coverslips, in a myocyte culture medium composed as follows: medium 199, 2g/L BSA, 2% FBS, 2mM Carnitine, 5mM Creatine, 5mM Taurine, 1mM Butanedione and antibiotics (all from Sigma-Aldrich), pH=7.35, and incubated for 1hr at 37°C, 5% CO_2_ to allow attachment to culture dish. Then culture medium was replaced with fresh one and cells were incubated for 4 more hours.

Next, medium was replaced by cardiomyocyte culture media mixed with concentrated conditioned media collected from SDF-1^non^ or SDF-1^mig^ or un-fractioned BM-MNCs or unconditioned EBM-2 basal medium as control. Synthetic angiogenin (BD, 10ng/ml) was added to the medium as positive control. For the experiments of inhibition, a recombinant protein of human ribonuclease/angiogenin inhibitor 1 (RNH1, OriGene) was added to SDF-1^mig^ BM-MNCs conditioned medium at a concentration based on a stoichiometric ratio of 2:1 with average angiogenin concentration in conditioned media, 1hr prior to incubation on the cells.

Cardiomyocytes were exposed to hypoxia (2% O_2_, 37°C, 5% CO_2_) for 15 hrs. At the end of this period, cells were washed with PBS and fixed with 4% PFA for 15 minutes at RT, afterward PFA was replaced with -20°C cold methanol for 10 min at RT, to allow the cell permeabilization. For immuno-staining, cells were washed with PBS and incubated with a blocking solution (5% Horse Serum in PBS) for 45 min at RT. Then cells were incubated for 16hrs at 4°C with a primary antibody that recognizes the stress granules marker eIF3 (Santa Cruz, goat polyclonal, 1:200 in 5% Horse Serum in PBS), followed by incubation with an Alexa555 donkey-anti-goat secondary antibody (Invitrogen, 1:200 dilution in PBS, 1hr RT). Finally, cells were washed with PBS and mounted with a mounting solution added of 1μg/mL of DAPI for nuclei staining. Cells were analyzed at a X400 magnification. At least 600 cells were counted for each replicate. To test the purity of isolated cells, cardiomyocytes were permeabilized using Triton-X100 (0,1% in PBS, 10 min RT) and stained with an antibody anti-alpha sarcomeric actinin (Sigma, mouse monoclonal, 1:500 dilution, 1hr 37°C) followed by incubation with an Alexa488 goat-anti-mouse secondary antibody (Invitrogen, 1:200 dilution in PBS, 1hr RT). Experiments were performed in duplicates using the conditioned medium from BM-MNC of 3 donors;

**Assessment of infarct size and interstitial fibrosis**

Infarct size and interstitial fibrosis in the peri-infarct myocardium were analyzed by Azan Mallory staining.^1^ For the assessment of infarct size, images of the entire LV were taken at a 12.5X magnification in n=3 not consecutive sections of individual hearts. For the assessment of interstitial fibrosis, 10 fields were evaluated in each section at X200 magnification. The *Image J* software was employed to calculate the percentage of LV area covered by the fibrotic scar.

**Assessment of cardiomyocyte proliferation and apoptosis.**

Immunohistochemistry analysis was performed on left ventricular cryosections (8 μm thick) post-fixed with ice-cold acetone (Sigma-Aldrich, Dorest, UK) for 10 min, and rinsed with PBS. For analysis of proliferation, *n*=3 sections were permeabilized with 0.1% Triton X-100 in PBS, for 10 min at RT, and incubated with rabbit polyclonal Ki67 antibody (Abcam, UK, 1:100, O.N. 4°C), followed by goat-anti rabbit secondary antibody conjugated with Alexa Fluor 488 (Invitrogen, UK, 1:200, 1h RT), and mouse monoclonal α-sarcomeric actin (Sigma, 1:200, 1h 37°C) followed by goat-anti mouse secondary antibody conjugated with TRITC (Invitrogen, UK, 1:200, 1h RT). Nuclei were recognized by DAPI staining. The data were expressed as fraction of Ki67^pos^ myocyte nuclei in the peri-infarct zone. Cardiomyocyte apoptosis was quantified by the terminal deoxynucleotidyl-transferase (TdT)-mediated dUTP nick-end labeling (TUNEL) technique (in situ cell death detection kit Fluorescein, Apoptag, Millipore, Germany). Following the treatment of n=2 slides with proteinase K (Sigma, 15μg/ml, 10min at RT), the TUNEL assay was performed according to the manufacturer’s instructions. Finally sections were stained with DAPI to recognize nuclei. The data were expressed as fraction of TdT^pos^ myocytes nuclei in peri-infarct zone. Sections were analysed at a 400X magnification. Adobe Photoshop software was utilized to compose and overlay the images (Adobe).

**Vascular density profiling**

For capillary density, 10 µm thick LV cryosections were incubated with biotinylated Isolectin B4 (Invitrogen, UK, 1:200,16 hrs at 4°C in a humidified chamber), followed by streptavidin Alexa Fluor 488 (Invitrogen, UK, 1:100, 1h at room temperature). For arteriole density, the same sections were probed with anti-mouse α-smooth muscle cell actin antibody conjugated with Cy3 (Sigma chemicals, UK, 1:400, 1h at room temperature). Capillaries and arterioles were calculated in 8 fields at X400 magnification and the final data expressed as the number of capillaries or arterioles per square millimetre. Arterioles were also categorized according to their luminal size.

**Quantitative PCR analysis of Angiogenin**

Total RNA from BM-MNCs was extracted and purificated using the miRNeasy Mini Kit (Quiagen) as per manufacture’s instructions. Extracted total RNA was reverse-transcribed into single-stranded cDNA using a High Capacity RNA-to-cDNA Kit (Life Technologies). The RT-PCR was performed using first-strand cDNA with TaqMan Fast Universal PCR Master Mix (Life Technologies). The assay numbers for the endogenous controls and target genes were as follows: *UBC* (Hs00824723_m1) and *Angiogenin (Hs04195574_sH).* Quantitative PCR was performed on a LightCycler480 Real-Time PCR system (Roche Technologies). Quantitative PCR parameters for cycling were as follows: 50° C incubation for 2 min, 95° C for 10 min, 40 cycles of PCR at 95° C for 15 s, and 60° C for 1 min. All reactions were performed in a 10 μL reaction volume in triplicate. The mRNA expression level was determined using the 2^−Δ^*^C^*^t^ method.

**Table S1: Key inclusion criteria of TransACT trials.**

| **Trial** | **Inclusion criteria** |
| --- | --- |
| ***TransACT 1 (phase II trial*)** | Age 16 to 80 years |
|  | Recent (>10 days and 3 months) large ST-Segment Elevation Myocardial Infarction (STEMI) |
|  | Absence of severe LV dilatation (LV end-systolic volume index <60 ml/m^2^) |
|  | MRI documented MI in one coronary territory of >/= 50% wall thickness in at least 1 segment |
|  | Indication for coronary artery bypass grafting surgery |
| ***TransACT 2 (pilot trial)*** | Age 16 to 80 years |
|  | Previous myocardial infarction (with evidence of large surgically excludible scar at cardiac MRI) |
|  | Left ventricular ejection fraction ≤ 35% |
|  | NYHA class III/IV and one episode of congestive heart failure |
|  | Indication for elective left ventricular restoration surgery |

**Table S2**: **Basic cardiovascular risk profile of BM donors** (Quantitative data are expressed as number or as median with minimum and maximum; n=24)

| Age (y) | 66±2 |
| --- | --- |
| Male/Female | 22/2 |
| Diabetes | 3 |
| Smoking:  Current  Ex smoking >1month | 20  13  7 |
| Hypertension | 16 |
| Hypercholesterolaemia | 24 |
| Previous MI:  Recent (TransACT1)  Chronic (TransACT2) | 24  18  6 |

**
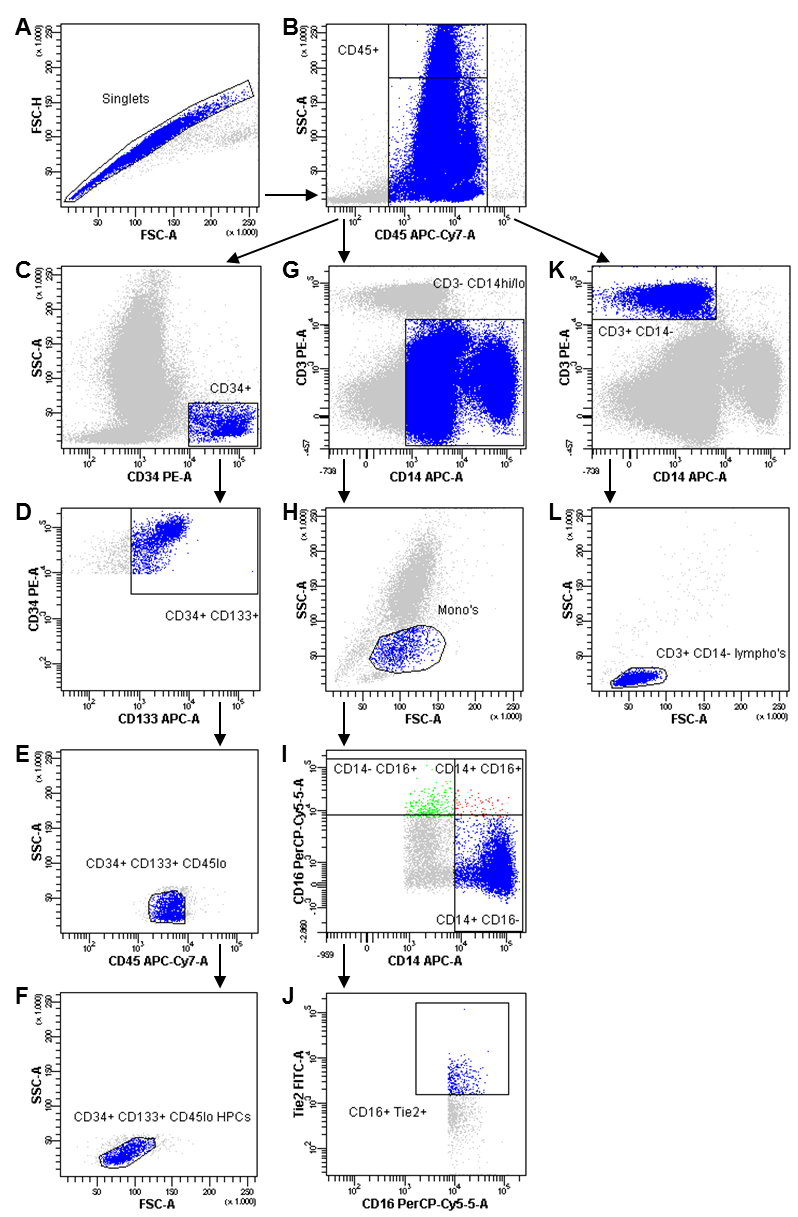
**

**Figure S1: Gating strategy for antigenic characterization of BM-MNCs by flow cytometry. A-F**, hematopoietic progenitor cells (HPCs). **A, B & G-J**: monocytes.  **A, B, K & L**: lymphocytes.

**
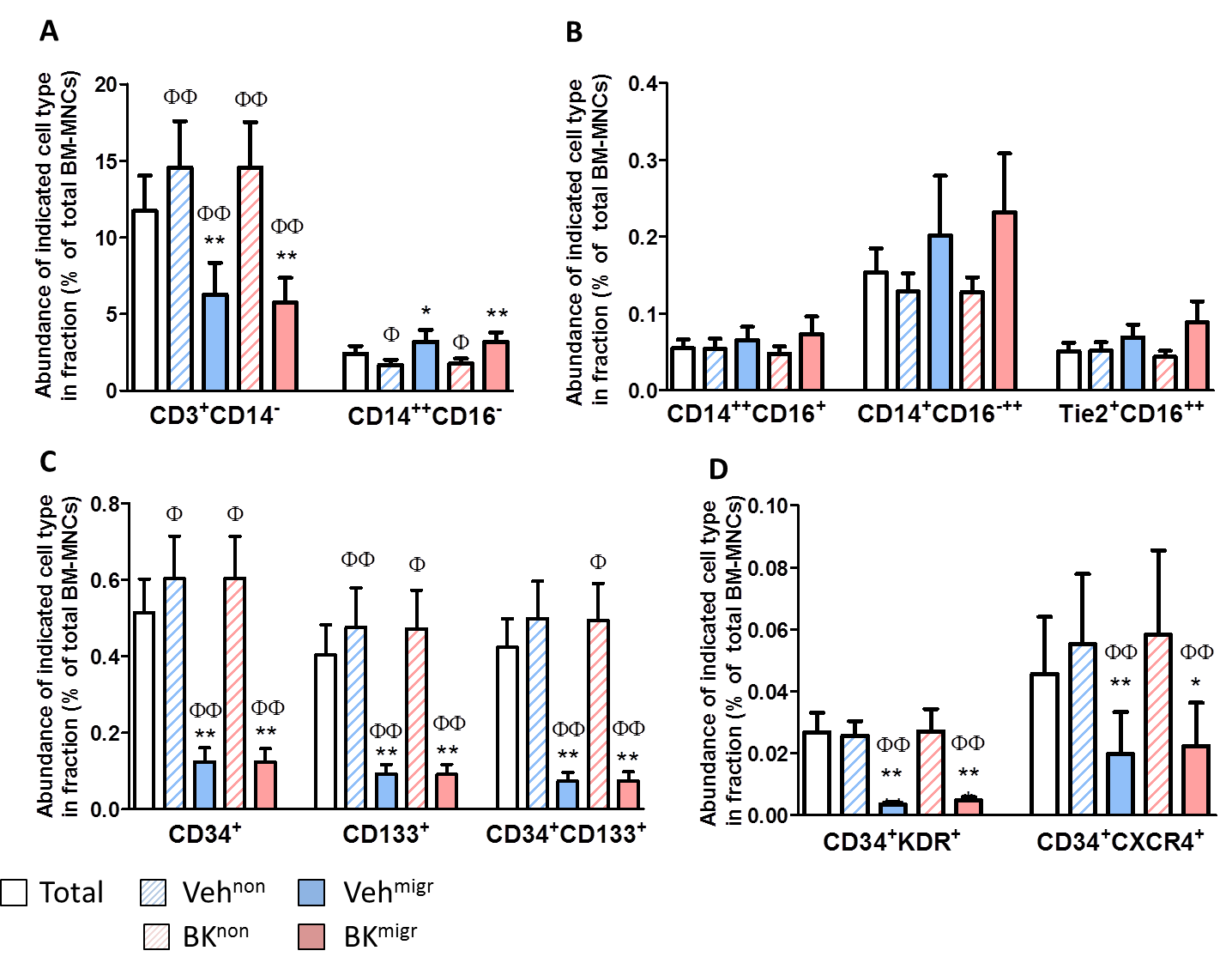
**

**Figure S2: *In vitro* migration towards bradykinin alters the composition of BM-MNCs. A-D,** Bar graphs show the percentage of lymphocytes and classical monocytes (**A**), intermediate and non-classical monocytes (**B**) and progenitor cells (**C&D**). Values are means±SEM, n=10 in each group. ^Φ^*P*<0.05 and ^ΦΦ^*P*<0.01 vs. total BM, **P*<0.05 and ***P*<0.01 vs. corresponding non-migrated group.


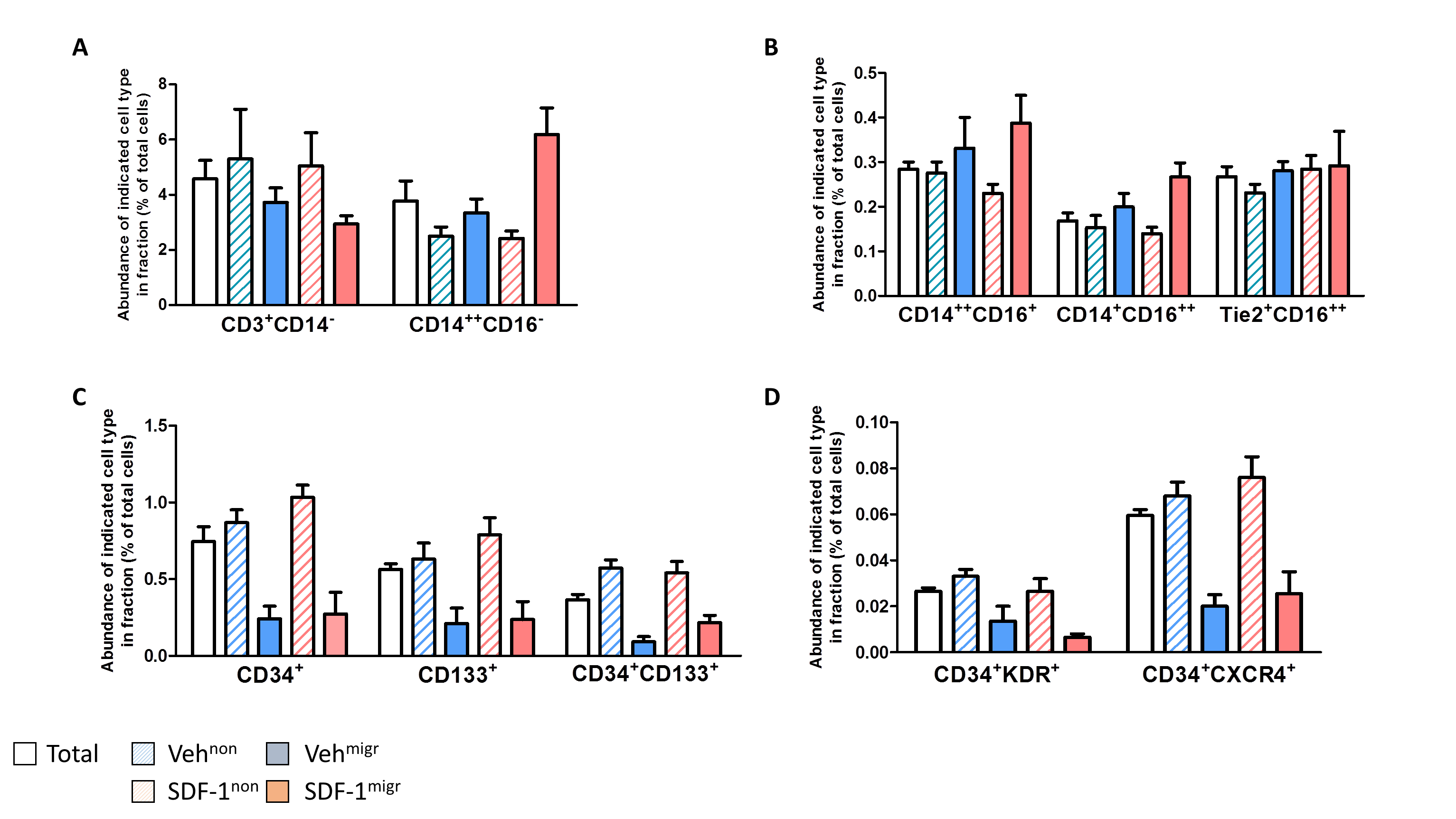


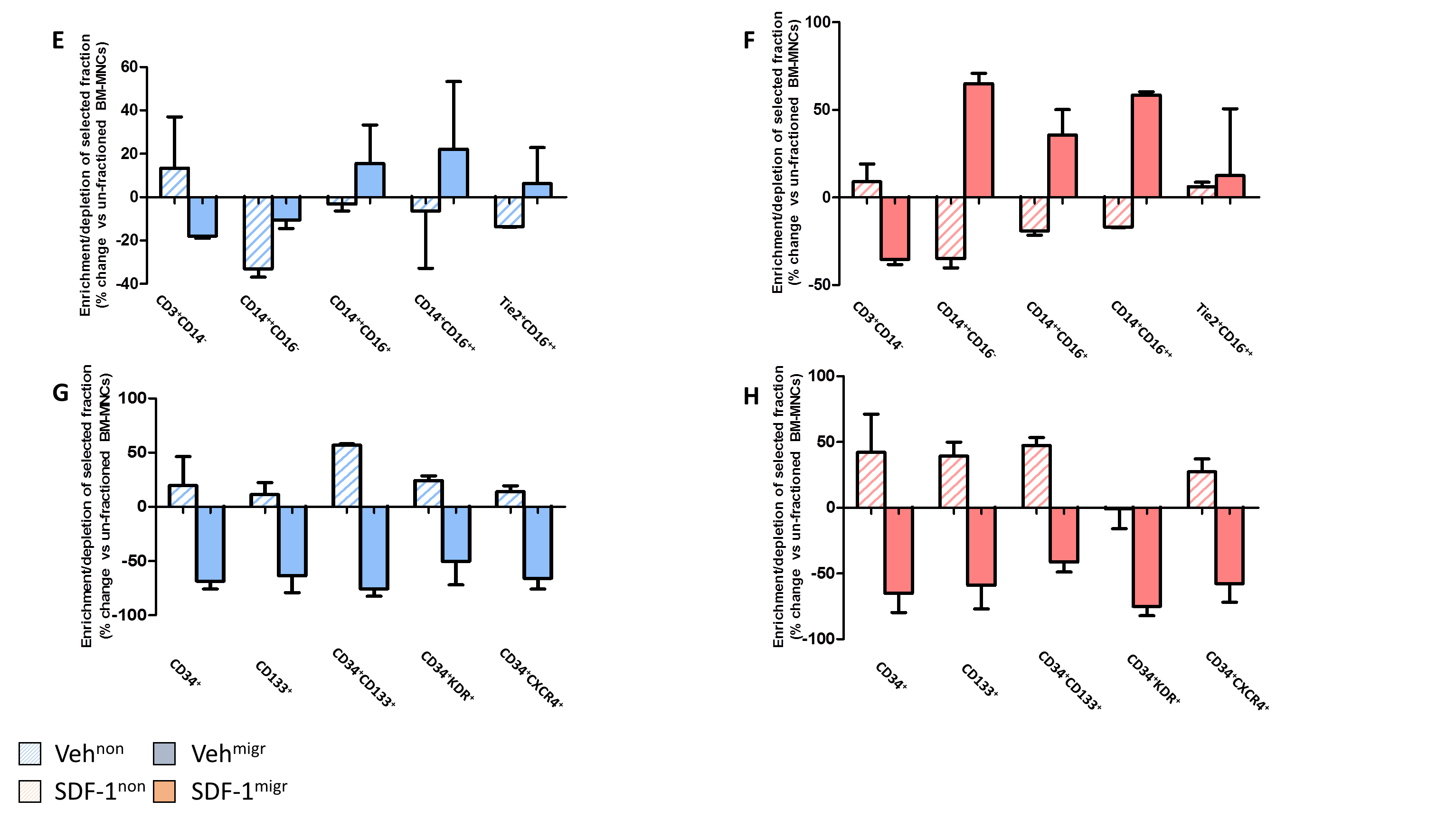


**Figure S3: *In vitro* migration of BM-MNCs from subjects without cardiovascular disease**. **A-D**, Percentage of lymphocytes and classical (**A**), intermediate and non-classical monocytes (**B**) and progenitor cells (**C&D**). **E-H**, Enrichment/depletion of antigenically-defined cell populations in migrated and non-migrated fractions following exposure to SDF-1 (**F&H**) or vehicle (**E&G**). Values are means±SEM of 2 biological replicates.


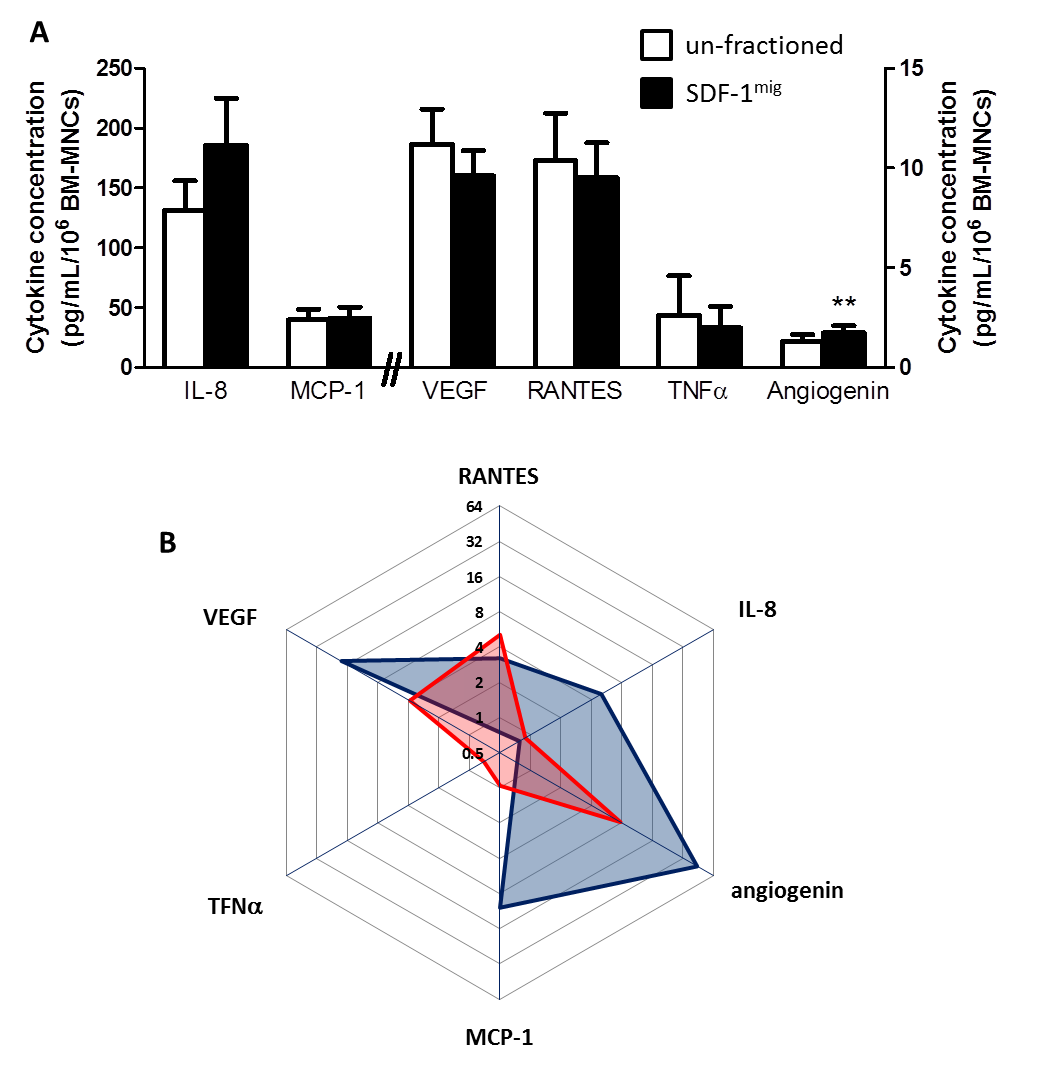


**Figure S4:** **Paracrine activity of BM-MNCs**. **A,** Bar graph shows the levels of cytokines in conditioned media collected from un-fractioned and SDF-1^mig^ cells. BM-MNCs were obtained from 10 donors for matched comparisons. Only factors expressed above the assay detection limits are shown. Values are means±SEM; ***P*<0.01 vs. un-fractioned. **B,** Radar plot shows the relation of secreted cytokines and capacity of BM-MNCs to promote network formation *in vitro*. Values in the radial scale represent F-to-enter (t^2^) from the regression analysis. Lines connecting F values delimit areas (blue for un-fractioned and red for SDF-1^mig^ cells) which illustrate the cumulative impact of secreted cytokines on the *in vitro* angiogenic process.


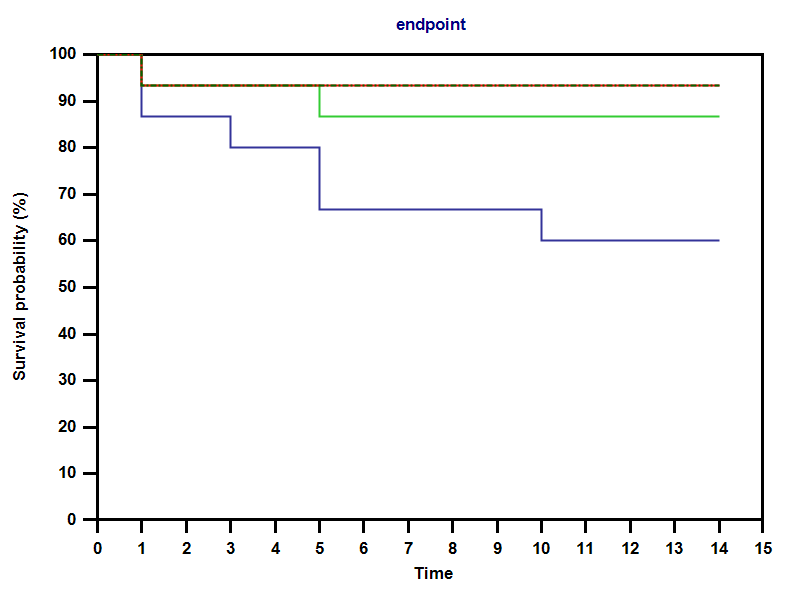


Survival probability

Time

**Vehicle**

**Un-fractioned**

**SDF-1 ^non^**

**SDF-1 ^migr^**

**Figure S5:** Kaplan-Maier survival curves in MI mice intra-myocardially injected with un-fractioned BM-MNCs, migrated (SDF-1^migr^) or non-migrated (SDF-1^non^) fractions. Control mice received vehicle (saline) instead of cell therapy.
